# Food Flavor Chemistry and Sensory Evaluation

**DOI:** 10.3390/foods13050634

**Published:** 2024-02-20

**Authors:** Diana De Santis

**Affiliations:** Department for Innovation in Biological, Agro-Food and Forest Systems DIBAF, University of Tuscia, 01100 Viterbo, Italy; desdiana@unitus.it

## 1. Introduction

The chemical composition of food plays a crucial role in determining its characteristics and properties. Modern analytical techniques have made it possible to identify and quantify the different chemical components present in food. These techniques allow us to measure the exact amounts of various nutrients, vitamins, minerals, volatile compounds, and other compounds in a given food sample. By analyzing these data, we can predict how these components may interact with each other and affect a food’s overall properties. This knowledge can benefit the food industry, helping to create products with specific nutritional profiles or other desirable characteristics. Additionally, it can aid in designing diets that meet specific health goals or dietary restrictions.

The nutritional and functional properties of food are two of the most important factors, but the sensory aspect cannot be overlooked. A vast number of substances interact with our chemical senses and influence the acceptability and pleasantness of foods.

Food products contain many volatile compounds that contribute to their odor and flavor. These represent two of the main factors that influence consumer choices.

Therefore, the chemistry of food flavor is a topic of great interest in food research due to its potential to impact the commercial success of products. 

This makes chemical identification and sensory evaluation essential in food research and product development projects. Recent advancements in analytical techniques and the ability to combine different chemical–sensory approaches have led to an exciting line of research that is exploring the relationships between flavor chemistry, sensory profiles, and food consumer preferences. 

So far, numerous techniques have been applied to identify volatile components in foods.

In recent years, many research works have attempted to develop analysis methods capable of chemically identifying aromatic profiles by seeking valid relationships with sensorial perception and consumer acceptability, adopting classic or innovative techniques such as headspace gas chromatography/mass spectrometry (HSGC-MS), two-dimensional gas chromatography mass spectrometry (GC×GC-MS), headspace gas chromatography–ion mobility spectrometry (GC-IMS), gas chromatography-olfactometry (GC-O), odor activity value (OAV), and combinations of these [1,2,3].

Although much research has been conducted on aroma, most of it is limited to studying specific plant and animal foods, both those that are traditional in particular geographical areas and those that are more widely distributed [4,5,6,7].

## 2. An Overview of Published Articles

Being of great relevance due to the multiple relationships with the quality, authenticity, and acceptability of foods, amongst other factors, the chemical and sensorial study of aroma has been identified as the topic of this Special Issue entitled “Food Flavor Chemistry and Sensory Evaluation”.

This Special Issue has collected 12 high-quality manuscripts that skillfully use innovative and conventional techniques to explore these aspects of various traditional and innovative food products, providing valuable information to the industry.

The research areas covered in these articles are diverse and range from exploring the chemical composition of the flavor profile of different food matrices to studying changes in flavor due to processing, storage times, and other factors.

Some papers use advanced predictive analytical techniques to study aroma, while others employ multiple analytical approaches to characterize it. These approaches include gas chromatography–mass spectrometry (GC-MS), E-Nose, GC-Quadrupole MS, GC-Orbitrap-MS, and sensory evaluation.

The studies also cover a wide range of food matrices, including fruits, vegetables, wines, oils, and baked goods.

Overall, the articles in this Special Issue provide a broad overview of the current state of aroma research, highlighting the importance of aroma in foods and the various approaches used to study it.

The study of Yuan Guo et al. [contribution 1] aimed to explore tissue-specific variations in the volatile flavor profiles of the *Lentinula edodes* (shiitake mushroom) fruiting body. Specifically, the research investigates the volatile compounds in different tissues, including the pileus skin, context, gill, and stipe, of two widely cultivated *L. edodes* strains (T2 and 0912).

Crucially, the study introduces machine learning analysis, demonstrating that prediction accuracy for different strains and tissues based on volatile profiles can reach an impressive 100%. This underscores the distinct strain- and tissue-derived volatile variations. The findings emphasize the need to consider both strain and tissue differences as essential variables when developing products with specific volatile flavor characteristics.

In summary, the research provides novel insights into the tissue-specific volatile flavor variations in the *L. edodes* fruiting body, shedding light on the significance of strain and tissue differences in the development of products with desired volatile flavor characteristics.

Rutkowska et al. [contribution 2] investigated storage-related changes in the volatile profiles and sensory properties of cookies containing xylitol as an alternative to sucrose. Xylitol offers a potential healthier substitute for sucrose, high-fructose corn syrup, and synthetic additives in industrial production, providing an alternative with potential health benefits, thanks to its lower glycemic index and caloric value. Using GC-MS with solid-phase microextraction (SPME) and quantitative descriptive analysis, the research reveals similar volatile compound profiles in both xylitol and sucrose biscuits, particularly regarding markers of the Maillard reaction and unwanted compounds.

Xylitol was found to contribute to improved pH, water activity stability, and sensory attributes, such as a buttery aroma and desirable texture characteristics, over a 12-month shelf life. However, the results suggest that a maximum shelf life of 9 months is recommended for these cookies.

The chemical and sensory differences between Marselan and Cabernet Sauvignon dry red wines in China were investigated by Xixian et al. [contribution 3], using GC-MS and high-performance liquid chromatography–triple quadrupole mass spectrometry (HPLC-QqQ- MS/MS).

In summary, this research has provided exciting and comprehensive insights into the chemical and sensory distinctions between Cabernet Sauvignon and Marselan wines in China, shedding light on the critical compounds that influence aroma, color, and tannin quality, as well as unique sensory attributes. It underlines how the multi-analytical approach can reveal exciting information on differences in flavor characteristics.

The study conducted by Niimi et al. [contribution 4] is groundbreaking research that adopted machine learning algorithms to predict sensory attributes in food products. Specifically, the research employs the extreme gradient boosting (XGBoost) method, a powerful machine learning technique for prediction modeling. The study focuses on wine sensory attributes during the grape-to-wine transformation process. The researchers aimed to predict twenty-two sensory attribute scores from five sensory stimuli, including aroma, color, taste, flavor, and mouthfeel. To do so, the researchers used absorbance–transmission and fluorescence excitation–emission matrix (A-TEEM) spectra from grape extracts to build a prediction model.

In summary, the research introduces a novel approach utilizing machine learning and fused spectral data to predict sensory attributes in the grape-to-wine transformation process, showcasing potential applications in the broader agri-food sector and transformed food products.

Sensory studies characterizing the aromatic profile of food products are frequent. Tura et al. [contribution 5] defined the flavor profile of cold-pressed hemp (*Cannabis sativa* L.) seed oil (CP-HSO), which has gained popularity due to its properties and nutritional profile. Seed quality, processing methods, and storage conditions influence the sensory quality of CP-HSO. Sixteen commercial CP-HSOs representing various brands and sales channels were evaluated in this study.

The sensory profile of cold-pressed hemp oil was drawn up by a group of trained tasters who identified 44 attributes and grouped them into clusters for both positive aspects and defects.

This study marks the first effort to standardize the sensory quality and terminology of CP-HSO, providing a comprehensive framework for evaluation and description in the industry.

The volatile profiles of *Panax ginseng* (Korean ginseng) and *Panax quinquefolium* (American ginseng) grown for different cultivation years were investigated by Kim et al. [contribution 6]. Using HS-SPME/GC-MS and chemometric analysis (PCA, HCA, PLS-DA), 56 compounds were identified in ginseng roots, including terpenes, alcohols, alkane, ketone, and furan. Chemometric analysis revealed distinct clusters for American and Korean ginseng cultivars based on volatile compositions. Terpenes, especially panaginsene, ginsinsene, α-isocomene, and caryophyllene, were predominant in Korean ginseng, while β-farnesene levels were higher in American ginseng. Differences in sesquiterpene composition, including β-panaginsene, ginsinsene, caryophyllene, and β-farnesene, contributed to the variations in volatile patterns between the two ginseng species.

Similarly, to explore the chemical composition of aroma and aromatic characteristics, Liu et al. [contribution 7] investigated passion fruit wines using a gas chromatograph quadrupole mass spectrometer (GC-qMS), GC-Orbitrap-MS, electronic nose (E-nose), and sensory evaluation. The E-nose confirmed distinct aromatic features in these wines. GC-Orbitrap-MS detected 17 sulfur compounds and GC-qMS identified 78 volatiles, with 44 significantly contributing to the overall wine aroma. Partial least squares regression indicated correlations between sulfides, esters, and terpenes with specific fruit aromas. Sulfur compounds significantly influenced the aroma of passion fruit wine, providing valuable insights for quality control.

Escott et al. [contribution 8] explored the synergistic effect of *Metschnikowia pulcherrima* and *Lachancea thermotolerans* on acidification and aromatic compounds in Airén wines. Ternary fermentations with these species helped to find increased ethyl lactate and 2-phenylethyl alcohol concentrations with M. pulcherrima and elevated lactic acid production with *L. thermotolerans*. Lower pH levels and reduced chemical oxidation were observed, and the wines received higher overall ratings in terms of sensory evaluation. Ternary fermentations of these non-*Saccharomyces* species are suggested as an alternative to spontaneous fermentations, enhancing freshness in wines from neutral varieties.

Yakub et al. [contribution 9] evaluated the effects of odor on fortifying vanilla milkshakes with microencapsulated microalgae oil (S17-P100). A 10-step oil enrichment protocol was developed, resulting in a robust recovery of docosahexaenoic acid (DHA). The flash-GC-based E-nose was used to assess odor profiles, and chemometric analyses were performed. While differences in odor were observed between the control and supplemented products, no systematic pattern corresponding to supplementation percentages was identified. The study suggests that no detectable off-odor resulted from increased oil concentration, with specific volatile compounds present in the supplemented product. The findings emphasize the potential for efficient fortification without compromising sensory quality.

The study of Aung et al. [contribution 10] explored the flavor profile of hot and cold roasted–steamed–germinated wheat beverages using electronic sensors and chemical analysis. Different concentrations of hot and cold beverages were analyzed for amino acids, volatiles, taste, total flavonoid content, total phenolic content, and antioxidant capacities. Statistical analyses, including correlation patterns, clustering, and principal component analysis, revealed significant differences between hot and cold beverages. The study demonstrates the impact of temperature on the aroma metabolites, taste, and characteristics of wheat beverages.

Sensomics approaches were used by Wang et al. [contribution 11] to characterize the aroma of peach spirits produced via distillation and pervaporation. Pervaporation technology is applied for the first time, and critical aromatic compounds are compared. Pervaporation-produced peach spirits exhibit stronger fruity, honey, and acidic aromas. Aroma-active regions are identified, with esters, lactones, and acids being significantly higher in pervaporation-produced spirits. The study identifies the critical aromatic compounds that can explain the differences in aroma profiles between the two types of peach spirits.

Li et al. [contribution 12] specified the dominant aromatic compounds in *Guangxi*-fermented bamboo shoots (GFBSs) using gas chromatography-olfactometry–mass spectrometry, odor activity values, and aroma recombination. Seventy aromatic compounds were identified, with 15 having significant odor activity values. Aroma recombination experiments highlighted the compounds contributing to the characteristic aroma of GFBSs, including p-cresol and acetic acid. The major aroma profile was described as a strong fermented odor with pungent and sour notes.

The results from the various studies will significantly contribute to the study of aroma, providing valuable insights into the chemical composition and perceived sensorial profile of different matrices. Using multi-analytic approaches to verify the differences and quality of the various matrices studied can help to achieve a complete understanding of the topic.

The complexity of aroma research is reflected in the presented works, highlighting the importance of a multidisciplinary approach that uses conventional and innovative techniques. These approaches can help researchers to better understand and analyze the various factors that influence aroma, such as the origin of matrices, processing methods, and storage conditions.

Furthermore, the results of these studies may be helpful for industries involved in producing and processing food products. The findings can aid in the selection of better quality raw materials, as well as the development of improved processing techniques that can aid overall flavor expression.

In summary, the presented works provide a solid basis for future research in the study of aroma. Through a multidisciplinary approach, researchers can continue to explore the topic’s complexity and develop innovative techniques to better understand and improve the sensory experience of food products.

## Data Availability

No new data were created or analyzed in this study. Data sharing is not applicable to this article.
